# Integrating artificial neural networks, multi-objective metaheuristic optimization, and multi-criteria decision-making for improving MXene-based ionanofluids applicable in PV/T solar systems

**DOI:** 10.1038/s41598-024-81044-3

**Published:** 2024-11-27

**Authors:** Tao Hai, Ali Basem, As’ad Alizadeh, Kamal Sharma, Dheyaa J. jasim, Husam Rajab, Abdelkader Mabrouk, Lioua Kolsi, Wajdi Rajhi, Hamid Maleki, Narinderjit Singh Sawaran Singh

**Affiliations:** 1 Artificial Intelligence Research Center (AIRC), Ajman University, P.O. Box 346, Ajman, UAE; 2 Faculty of Data Science and Information Technology, INTI International University, Nilai, 71800 Malaysia; 3 Faculty of Engineering, Warith Al-Anbiyaa University, Karbala, 56001 Iraq; 4 Department of Civil Engineering, College of Engineering, Cihan University-Erbil, Erbil, Iraq; 5 Institute of Engineering and Technology, GLA University, Mathura, U.P 281406 India; 6 Department of Petroleum Engineering, Al-Amarah University College, Maysan, Iraq; 7 College of Engineering, Department of Mechanical Engineering, Najran University, King Abdulaziz Road, P.O Box 1988, Najran, Kingdom of Saudi Arabia; 8 Department of Civil Engineering, College of Engineering, Northern Border University, Arar, 73222 Saudi Arabia; 9 Department of Mechanical Engineering, College of Engineering, University of Ha’il, Ha’il City, 81451 Saudi Arabia; 10 Laboratoire de Mécanique, Matériaux et Procédés LR99ES05, Ecole Nationale Supérieure d’Ingénieurs de Tunis, Université de Tunis, 5 Avenue Taha Hussein, Montfleury, 1008 Tunis Tunisia; 11 Department of Mechanical Engineering, Isfahan University of Technology, Isfahan, Iran

**Keywords:** Artificial neural network, Multi-objective optimization, Multi-criteria decision-making, Solar energy, MXene-based nanofluid, PV/T system, Energy science and technology, Engineering, Materials science

## Abstract

Optimization of thermophysical properties (TPPs) of MXene-based nanofluids is essential to increase the performance of hybrid solar photovoltaic and thermal (PV/T) systems. This study proposes a hybrid approach to optimize the TPPs of MXene-based Ionanofluids. The input variables are the MXene mass fraction (MF) and temperature. The optimization objectives include three TPPs: specific heat capacity (SHC), dynamic viscosity (DV), and thermal conductivity (TC). In the proposed hybrid approach, the powerful group method of data handling (GMDH)-type ANN technique is used to model TPPs in terms of input variables. The obtained models are integrated into the multi-objective particle swarm optimization (MOPSO) and multi-objective thermal exchange optimization (MOTEO) algorithms, forming a three-objective optimization problem. In the final step, the TOPSIS technique, one of the well-known multi-criteria decision-making (MCDM) approaches, is employed to identify the desirable Pareto points. Modeling results showed that the developed models for TC, DV, and SHC demonstrate a strong performance by R-values of 0.9984, 0.9985, and 0.9987, respectively. The outputs of MOPSO revealed that the Pareto points dispersed a broad range of MXene MFs (0-0.4%). However, the temperature of these optimal points was found to be constrained within a narrow range near the maximum value (75 °C). In scenarios where TC precedes other objectives, the TOPSIS method recommended utilizing an MF of over 0.2%. Alternatively, when DV holds greater importance, decision-makers can opt for an MF ranging from 0.15 to 0.17%. Also, when SHC becomes the primary concern, TOPSIS advised utilizing the base fluid without any MXene additive.

**Supplementary Information:**

The online version contains supplementary material available at 10.1038/s41598-024-81044-3.

## Introduction

Recently, advancements in heat transfer enhancement techniques (HTETs) have been recognized as making a noteworthy contribution to thermofluids research. HTETs are oriented toward achieving objectives such as enhancing the efficiency of thermal devices, decreasing energy consumption, and compacting systems^1,2^. Approaches like dispersing nanomaterials (NMs) in fluids^3^, attaching extended surfaces^4–8^, incorporating swirl flow devices^9,10^, and adding porous materials^11^, are recognized as passive HTETs. They have garnered increased approval across different industries because of their practical implementation simplicity and satisfactory efficiency. As initially proposed by Choi^12^, the nanoparticle dispersion in fluids has brought about significant advancements in enhancing the thermophysical properties (TPPs) of fluids. This groundbreaking development has led to the emergence of nanofluids, which now play a pivotal role in diverse applications, including electronic cooling^13^ and biomedicine^14^, heat exchangers^15^, fuel cells^16^, solar energy^17^, lubrication^18^, thermal energy storage^19^, drug delivery^20^, and many others^21–24^.

The dispersion of nano-sized materials significantly affects the base fluid’s thermophysical, rheological, and tribological behaviors. Various types of research confirm that surfactants, concentration of NMs, type of NMs (shape and size), and temperature strongly affect specific heat capacity (SHC), stability, dynamic viscosity (DV), and thermal conductivity (TC) of base fluids, characteristics that determine the application of nanofluids in various industries^25–29^. Meanwhile, the type of NMs has the most significant effect on the discussed properties and, as a result, their application.

The importance of NMs led to efforts to discover high-efficiency materials in specific applications. With the discovery of 2D NMs such as graphene-based materials, boron nitrides, metal nanosheets, and transition metal oxides/dichalcogenides, a tremendous change occurred in nanotechnology research^30–33^. Graphene-containing composites have shown significant potential in many applications by showing excellent TPPs and acceptable stability^34–36^. However, disadvantages such as difficulty in mass production, environmental pollution, high price, and toxicity prevented its increasing development^37–39^, so the development of new 2D materials was placed on the agenda of many scientists in the nanotechnology field.

Naguib et al.‘s groundbreaking research in 2011 led to the discovery of an innovative transition metal material known as MXene^40^. MXene is synthesized through a selective etching process that involves removing the A layer from the MAX phases. This two-dimensional material exhibits properties similar to graphene, reduced graphene oxide, and graphene oxide^41^. MXenes offer exceptional mechanical, optical, and electronic characteristics, making them highly versatile and suitable for an extensive range of applications. These applications include ionic batteries^42^, energy storage^43^, biomedicine^44^, catalysts^45^, and sensors^46^. The exceptional tunability of MXenes’ structural flexibility and surface properties has opened up exciting possibilities across various fields.

In addition to the mentioned applications, concentrated photovoltaic/thermal (CPV/T) solar systems are one of the most important purposes of using MXene-based nanofluids. Various experimental and numerical studies have investigated the hydraulic and thermal behavior of nanofluids containing MXenes to make it feasible for CPV/T solar systems. In this regard, Wang et al.^47^ evaluated the photo-thermal conversion (PTC) and stability of MXene/water nanofluid in a direct absorption solar collector system. They found that the PTC efficiency in the presence of MXene-based nanofluid reaches a maximum value of 63.35%, which is 4.34% more than graphene-based nanofluid. By adding MXene nanomaterials to soybean oil, Aslfattahi et al.^48^ numerically examined the thermal behavior of a solar dish collector. The considered nanofluid with high TC and SHC had high potential for solar thermal applications. They reported that the system’s thermal efficiency in the presence of nanofluid is higher than that of pure fluid. Said et al.^49^ investigated a parabolic trough collector containing silicon oil/MXene (Ti_3_C_2_) nanofluid under 4E analysis. They found that the TC of the base fluid in the presence of MXenes can experience an increase of 70–89%. They reported the positive influence of nanofluid on the system in terms of environmental, economic, exergy, and energy.

Rubbi et al.^50^ experimentally investigated the Therminol55 oil containing MXene (Ti_3_C_2_) and CuO nanomaterials to increase the performance of the CPV/T solar systems. They measured the stability, optical, and thermophysical characteristics of the nanofluids. They found that the PTC of the system in the presence of MXene NMs improved significantly up to 85.98%. The increase in TC of TH-55/Ti_3_C_2_ and TH-55/Ti_3_C_2_-CuO nanofluids compared to the pure TH-55 was reported to be 84.55% and 80.03%. Sreekumar et al.^51^ numerically studied the effectiveness of a PV/T solar system in the presence of water/MXene nanofluid in terms of energy, exergy, environmental and economic aspects. Their results indicated that adding 2 wt% of MXene to water increases 17% and 3.5% for thermal and electrical efficiency, respectively. At the same concentration of NMs, the heat transfer coefficient showed an increase of 21.42%. They also claimed that it is possible to reduce the size of the system by 4.5–14.5% using MXene-based nanofluid. In another research, Kadirgama et al.^52^ measured the stability and optical properties of ethylene glycol (70%)/deionized water (30%)/MXene nanofluid as a promising candidate for solar applications. Their results demonstrated that the highest light absorption occurs at an MXene concentration of 1 wt%. They found the hydrophilic nature of MXene to be an excellent opportunity to produce highly efficient nanofluids for solar applications.

Jin et al.^53^ measured the TC of water/graphene and water/graphene/MXene nanofluids. They observed that graphene NMs increased the DV and TC of base fluid by 70.69% and 65.34%, respectively. TC of graphene/MXene/water nanofluid did not change much compared to graphene/water, while its DV showed a significant reduction. In a similar study, Mao et al.^54^ measured the TC of EG and water containing Ti_3_C_2_T_x_ MXene. Their outcomes indicated 27.3% and 30.6% improvement in TC for water/EG/MXene and water/MXene nanofluids. This increase in TC was while the DV experienced a slight increase. They predicted significant potential for MXene-based nanofluids for cooling applications. In addition, Bao et al.^55^ examined Ti_3_C_2_T_x_ MXene/EG nanofluid in terms of stability, TC, and DV. They reported up to 9.64% growth in TC for MXene-based nanofluid. DV of the developed nanofluid in a volume fraction of 1% was much lower than that of nanofluids containing graphene and multi-walled carbon nanotubes (MWCNTs) in a volume fraction of 0.1%. Also, the stability of EG/MXene nanofluid was observed in a period of 30 days, which showed excellent performance.

Artificial intelligence (AI) has attracted much attention as one of the leading phenomena of the last few years^56–58^. By imitating human intelligence and logic, AI has created a massive change in the world of science and technology. Combining human rationality with machine speed and accuracy has made understanding, planning, decision-making, and inference in complex processes with the highest efficiency^59^. Recently, neural networks and evolutionary algorithms have become one of the most effective modeling and optimization tools for highly intricate systems as part of artificial intelligence^60–62^. Nanofluids’ TPPs can pose a complex system when multiple input variables are involved, making them a serious challenge for accurate modeling and valid optimization^63–65^.

Accurate modeling of the TPPs of nanofluids can reduce the costs and time of frequent experiments. In recent years, artificial neural networks (ANNs) as a reliable tool in modeling extremely nonlinear systems have sidelined the traditional nanofluids’ TPP prediction techniques^66^. The ANNs have proven extremely effective in generating highly accurate predictions (R^2^ = 0.9981) for the TC of EG-based nanofluid including MWCNT-SiO_2_^67^. Furthermore, Esfe et al.^68^ have also developed an innovative ANN-driven model with an optimized structure, attaining an outstanding R^2^ value of 0.9989. In another research, a multilayer perceptron artificial neural network (MLPANN) was utilized for simulating various TPPs of water-based Fe_3_O_4_-coated MWCNT nanofluid. The outcomes were highly promising, revealing an impressive range of improved R-values between 0.9938 and 0.9999^69^. Chu et al.^70^ introduced an ANN-based model predicting the rheological properties of an oil-based nanofluid containing TiO_2_-MWCNT. Their ANN model predicted DV of nanofluid with high precision (R^2^ = 0.999). Due to the significant accuracy of ANNs, Sepehrnia et al.^71^ used this approach to model the DV of ZnO-MWCNT/oil nanofluid. They confirmed the high accuracy of the ANN-based model by reporting R^2^ = 0.9921.

Developed models for predicting TPPs in combination with an optimization algorithm can lead to optimal selection of operating parameters^72^. In this regard, many researchers have focused on TC and DV to optimize the properties of different nanofluids. Previous studies in this research area have employed various methods such as genetic algorithm (GA), response surface methodology (RSM), artificial neural networks (ANN), fuzzy logic (FL), particle swarm optimization (PSO), and non-dominated sorting genetic algorithm II (NSGA-II). Table 1 provides an overview of recent investigations of nanofluids optimization based on TC and DV.

**Table 1:** Recent studies on nanofluids optimization based on TC and DV.

Reference	Base Fluid	Nanomaterials	Modeling and Optimization Techniques
Esfe et al.^73^	Water/EG	Al_2_O_3_	RSM
Said et al.^74^	Water/EG	TiO_2_ and Al_2_O_3_	FL and PSO
Esfe and Tilebon^75^	Oil	Al_2_O_3_/MWCNT	RSM and NSGA-II
Maqsood et al.^76^	Oil	MWCNT	RSM and ANN
Amani et al.^77^	Water	MnFe_2_O_4_	ANN and GA
Amani et al.^78^	Water	clove-treated MWCNTs	ANN and GA
Esfe et al.^79^	Water	ZnO, SiO_2_, Al_2_O_3_, and CuO	RSM, ANN, PSO, and GA
Danish et al.^80^	Water	TiO_2_	RSM

According to the reviewed studies^48–50,53–55^, TC, DV, and SHC, as the most critical thermophysical properties, play a decisive role in nanofluids’ behavior in hybrid solar photovoltaic and thermal systems. The importance of optimizing the TPPs of nanofluids in PV/T systems led the present authors to develop a hybrid strategy based on machine learning (ML), multi-objective optimization (MOO), and multi-criteria decision-making (MCDM). This integrated framework, aimed at optimizing the TPPs, is distinct in that it adapts to different design scenarios for PV/T systems, enhancing the practical adaptability and accuracy of nanofluid performance. No comparable framework has been presented in previous works for the design of nanofluids in solar applications. Based on Table 1, prior studies have focused on the two-objective optimization of TC and DV. The present hybrid strategy expands this to include SHC as an important TPP in solar applications, offering a more comprehensive optimization specifically tailored for solar PV/T applications. For this purpose, a binary solution containing ionic liquid (IL) and Diethylene Glycol (DEG) with novel 2D nanomaterials (MXenes) is subjected to TPP optimization. These innovative Ionanofluids have not yet undergone optimization or modeling, enabling us to focus, for the first time, on MXene-based Ionanofluids with high-performance potential in this domain. The TPPs of this type of nanofluids are strongly dependent on MXene mass fraction (MF) and temperature. In the proposed hybrid approach, a well-known formula-based ML technique, the group method of data handling neural network (GMDH-NN), is used to model various TPPs in terms of input variables. The superior models for each TPP are then used as a basis for MOO by the newly incorporated multi-objective thermal exchange optimization (MOTEO) method, which complements the established and robust multi-objective particle swarm optimization (MOPSO) technique. Finally, by assigning an importance factor (weight) to each objective, the TOPSIS method, a well-known MCDM approach, selects the desirable optimal points according to diverse scenarios. Incorporating various design scenarios through the weighting of objectives is a novel approach within this field’s literature and represents an initiative in this research to advance design methodologies in this area. This approach allows for the integration of theoretical research into practical applications in solar PV/T systems, ensuring high reliability in the nanofluid development process. Furthermore, the process employed in this study has the potential to revolutionize not only the nanofluids field but also various engineering and industrial phenomena.

**Fig. 1:**
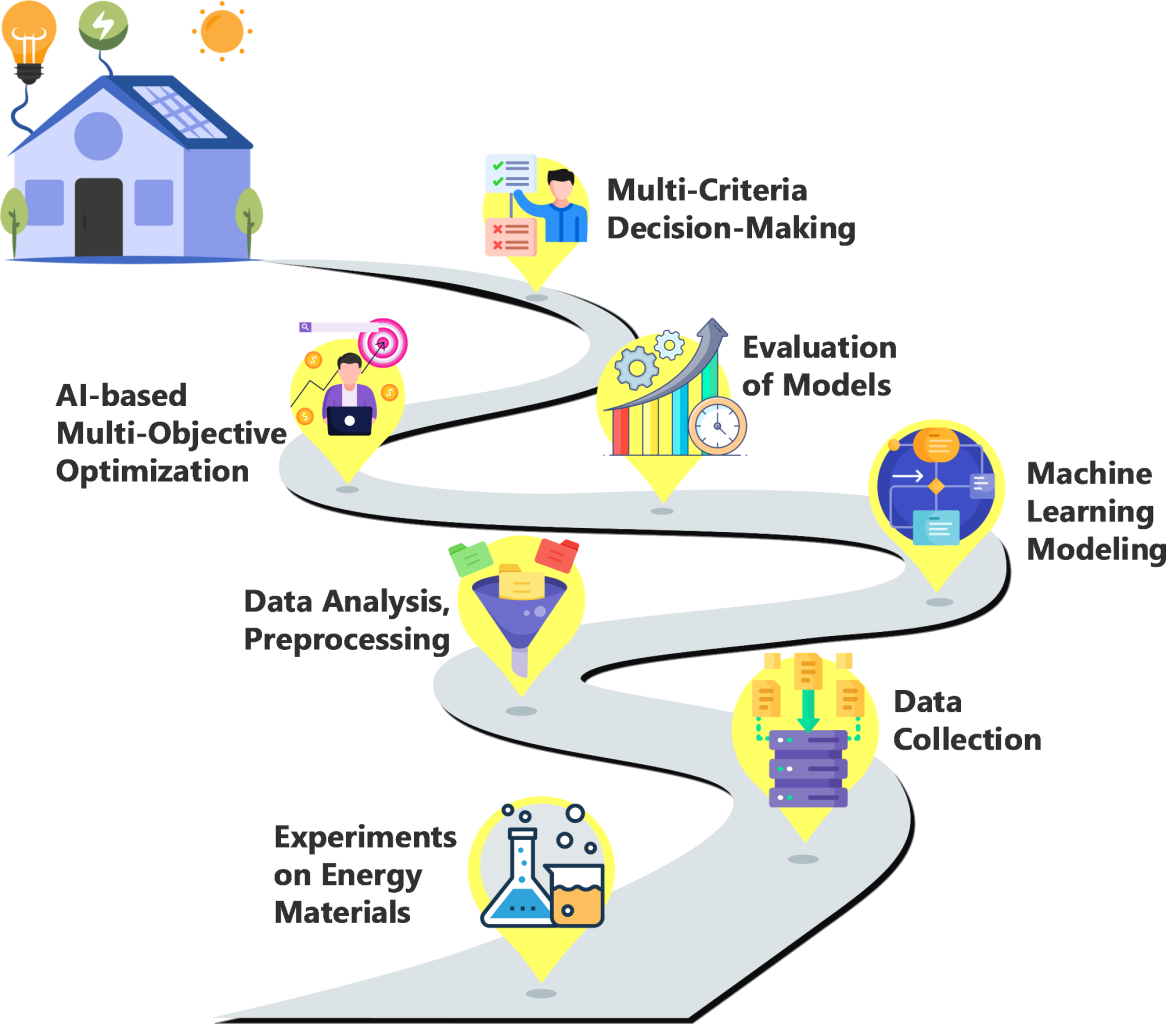
Schematic illustration of the proposed hybrid optimization process in the present study.

Figure 1 presents the proposed framework, outlining the process from experimental data to the optimal design of nanofluids aimed at enhancing the efficiency of solar-based renewable energy systems. The process begins by identifying a research gap concerning the thermophysical properties of high-potential nanofluids used in PVT systems, which have the potential to significantly enhance solar energy performance. The next step involves extracting and analyzing relevant data to gain a comprehensive understanding of the problem, as will be discussed in detail in Sect. 2. Following this, the data undergo machine learning modeling, where, in Sect. 3, GMDH-type neural network-based functions are developed to represent each of the three thermophysical properties as outputs in terms of input variables. Subsequently, these models are validated to assess their accuracy with unseen data. After confirming the robustness of the models, multi-objective optimization is conducted using MOPSO and MOTEO techniques in Sect. 4, treating the ANN models as objective functions. In Sect. 5, the optimal solutions obtained are further evaluated under various scenarios using the well-known TOPSIS method, enabling the selection of the most suitable solution through a systematic decision-making process. This approach supports the designer in making informed decisions to optimize PVT systems across different design scenarios.

## Database

This study uses the experimental datasets of Bakthavatchalam et al.^81^. By adding 2D MXenes (Ti_3_C_2_) to a binary solution containing ionic liquid and Diethylene Glycol, they produced a new nanofluid that improved the performance of hybrid photovoltaic/thermal systems to a great extent. They measured three critical characteristics, TC, DV, and SHC, in the MF range of 0-0.4 wt%. The temperature range for TC, DV, and SHC samples was 25–80 °C, 25–75 °C, and 20–80 °C, respectively.

Table 2 presents the descriptive statistics of TPPs in the mentioned MF and temperature ranges. Skewness and kurtosis, are considered to evaluate the deviation of the TPPs distribution from the normal (Gaussian) distribution. Divergence from the normal distribution can affect the complexity of the predictive models and their computational costs. According to previous research^82–84^, skewness values within the range of -1 to 1 and kurtosis values within the range of -2 to 2 are commonly used as benchmarks for assessing the normality of data distribution. According to Table 2, all three TPPs have a normal distribution regarding the skewness criterion. According to the kurtosis criterion, the SHC does not have a normal distribution, and the TC and DV are on the border of the normal distribution. Considering both criteria simultaneously, it can be stated that all three parameters have a distribution close to the normal distribution.

**Table 2:** Descriptive statistics of TPPs.

Descriptive Statistics	TC (W/m·K)	DV (mPa·s)	SHC (J/g·K)
Mean	0.5866	18.5048	2.4272
Std. Deviation	0.1492	8.5264	0.3699
Skewness	-0.2314	0.3595	0.4203
Kurtosis	1.9987	1.9840	3.0818

**Fig. 2:**
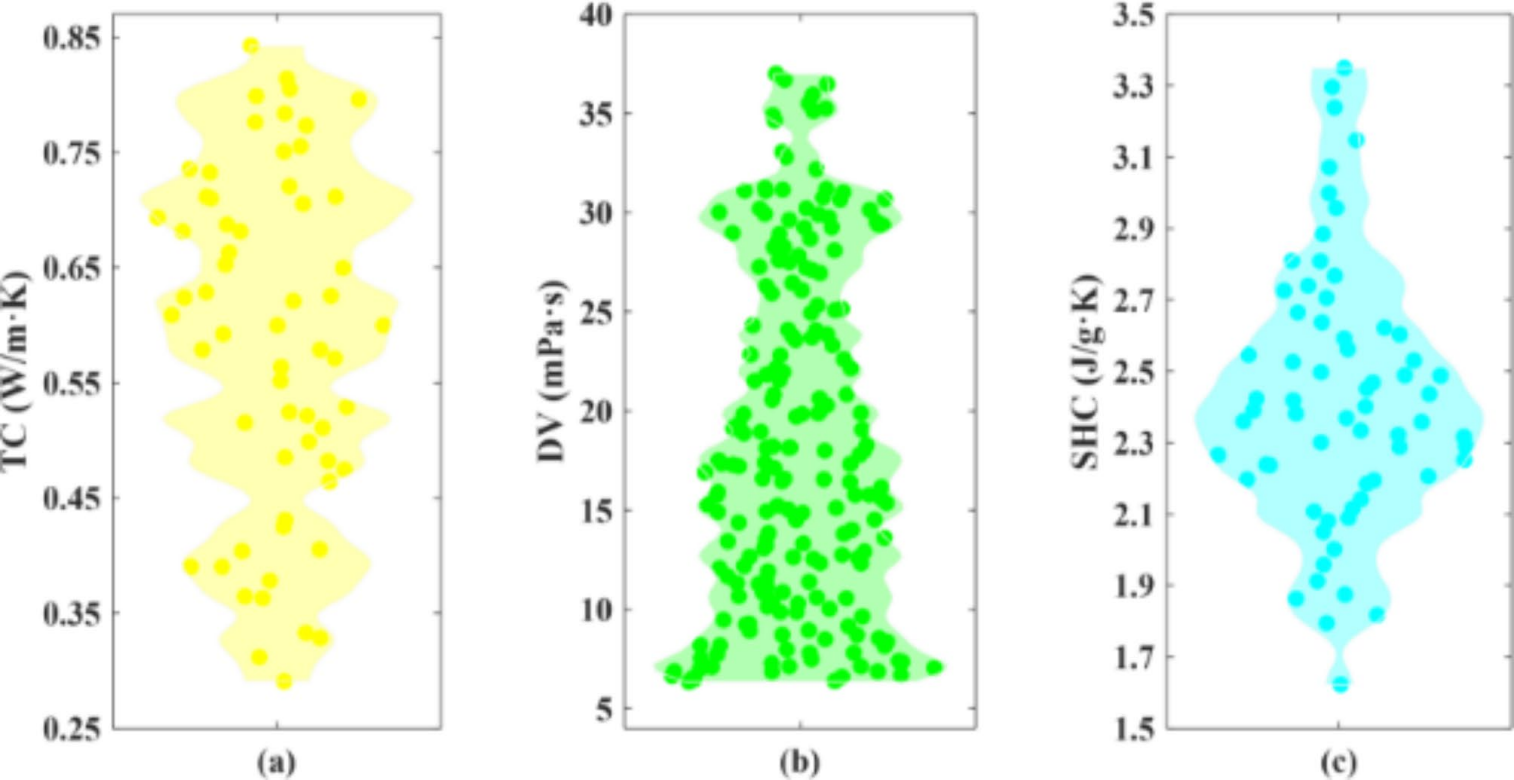
The violin plots of TPPs.

**Fig. 3:**
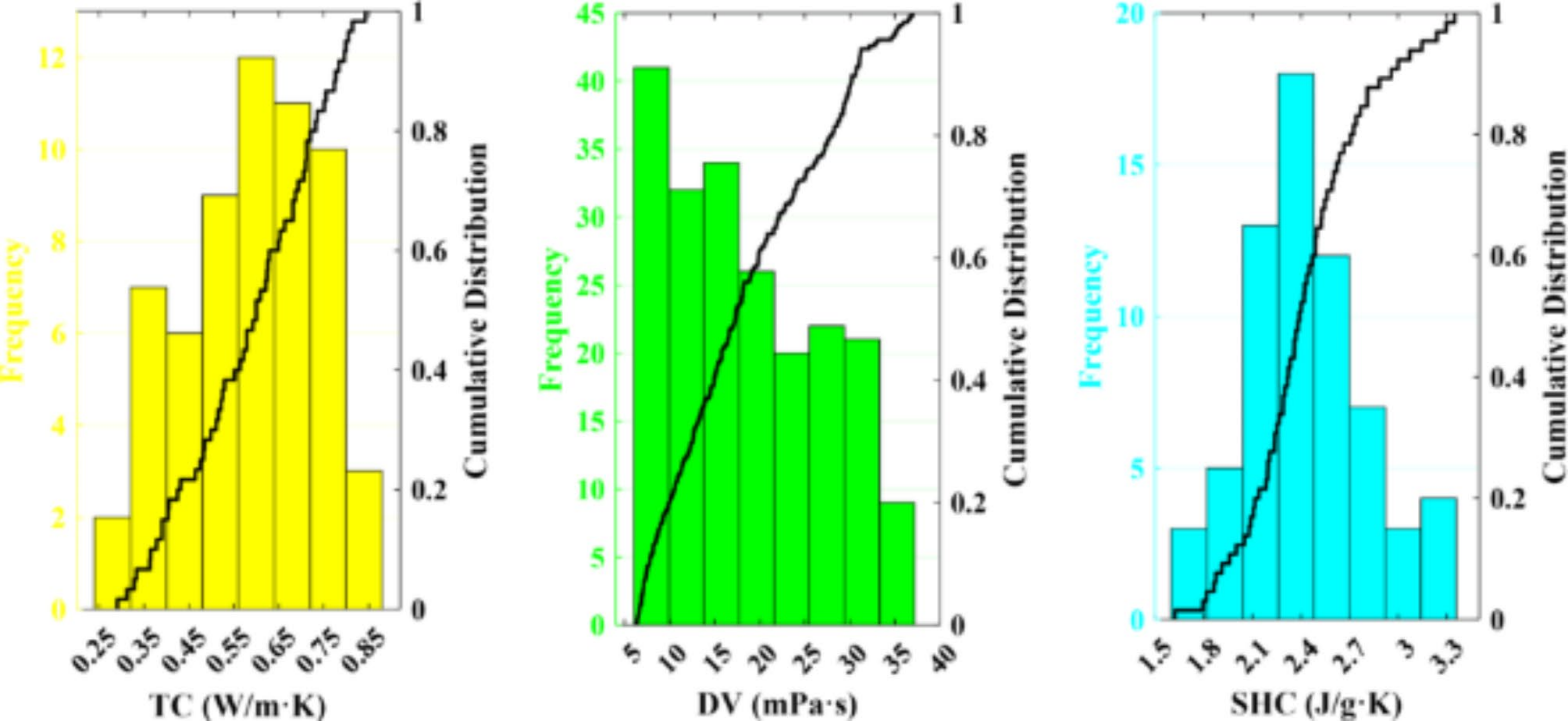
The frequency distribution histogram and cumulative distribution of TPPs.

Figure 2 shows violin diagrams for TC, DV, and SHC data points. In violin plots, the shape of the curves represents the probability density of the data at different values. The width of the curve indicates the frequency of data points at that particular value. The curves are generated using kernel density estimation (KDE), a statistical technique that estimates the probability density function of a random variable. Unlike histograms or box plots, which show counts or order statistics, violin plots focus on the shape of the data distribution. The dots in the violin plots are individual data points. Each dot represents an actual observation from the dataset. These points are plotted along the width of the violin, providing insight into the distribution of the data. The violin plot provides a convenient visual insight into peaks, positions, and relative amplitudes through data distribution and probability density. In addition, the frequency distribution histogram and cumulative distribution of TPPs are presented in Fig. 3. Figures 2 and 3 indicate the higher density of TC and SHC values near the mean value. While in the DV data points, the low values have more density.

Analyzing the linear relationship between inputs and outputs can provide a sufficient idea of the quality and quantity of the relationship between the variables. For this purpose, Pearson’s correlation coefficient (PCC) is one of the most well-known statistical measures. PCC accepts a value between − 1 and 1, the sign of which determines the direction of the relationship between the variables (quality), and its value determines the strength of their relationship (quantity). The tendency of the PCC to zero indicates that the variables are not related, and the tendency to 1 (or -1) indicates their strong relationship^85^. This criterion can be formulated as follows^86^:

1 $$PCC=\frac{\sum\:_{i=1}^{N}\left({X}_{i}-\stackrel{-}{X}\right)\left({Y}_{i}-\stackrel{-}{{Y}}\right)}{\sqrt{\sum\:_{i=1}^{N}{\left({X}_{i}-\stackrel{-}{X}\right)}^{2}\sum\:_{i=1}^{N}{\left({Y}_{i}-\stackrel{-}{{Y}}\right)}^{2}}}$$

where $N$ is number of data, ${X}_{i}$ and ${Y}_{i}$ are individual data points for variables X and Y, respectively. $\stackrel{-}{X}$ and $\stackrel{-}{{Y}}$ are the mean values of X and Y, respectively.

**Fig. 4:**
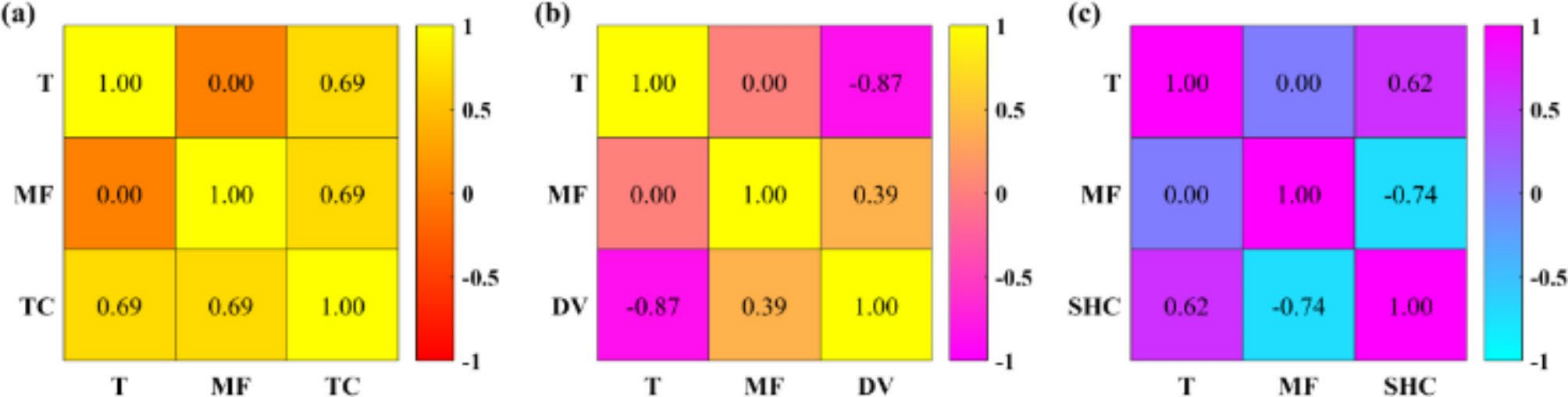
Pearson’s correlation coefficient diagrams for (a) TC, (b) DV, and (c) SHC.

Figure 4 displays Pearson correlation coefficient diagrams between TPPs and input variables. As can be seen, the same linear relationship is observed between TC and input variables in terms of quantity and quality (PCC = 0.69). However, DV is more linearly influenced by temperature (PCC = -0.87), and MF (PCC = 0.39) has less influence. On the other hand, the quantitative and qualitative relationship between SHC and input variables is opposite to that observed for DV. SHC is more affected by MF (PCC = -0.74), and temperature (PCC = 0.62) has a less positive linear correlation with SHC. It should be noted that, there may be a strong non-linear relationship between TPPs and input variables. Therefore, the weak correlation of some input variables with the TPPs should not be underestimated.

## Modeling

### GMDH-type neural network

ANNs represent a collection of computational models that have indicated remarkable potential in a broad spectrum of applications. They effectively emulate the intricate functioning of the human brain. One of the well-known types of neural networks is the group method of data handling (GMDH), devised by Ivakhnenko^87^, which was introduced to address certain limitations encountered in conventional neural networks. The utilization of feed-forward neural networks ensures stability throughout the modeling process. Furthermore, the GMDH-type ANN offers the advantage of reducing both the quantitative and qualitative reliance on the database. The principal benefit of this technique lies in its self-organization capabilities, enabling increased accuracy and reduced complexity of the final model through the elimination of underperforming sub-models (neurons)^64^. Notably, applying the GMDH approach for predicting intricate phenomena has witnessed substantial progress in recent years^60,88,89^.

In order to elucidate a system entailing $M$ data points and $n$ inputs, the utilization of a sophisticated function, denoted as $f$, becomes essential. This function possesses the capability to establish a connection between the inputs $x=\left({x}_{1},{x}_{2},\dots\:{x}_{n}\right)$ and the corresponding outputs ${y}_{i}$:

2 $${y}_{i}=f\left({x}_{i1},{x}_{i2},\dots\:{x}_{in}\right)\:\:\:\:\:\:\:\:(i=\text{1,2},\dots\:,M)$$

The primary goal of the GMDH approach is to establish a function, denoted as $\widehat{f}$, which can effectively forecast the outputs’ values. This goal is accomplished by reducing the disparity between the predicted values, referred to as ${\widehat{y}}_{i}$, and the actual values (${y}_{i}$). Mathematically, this purpose can be expressed as follows:

3 $${\widehat{y}}_{i}=\widehat{f}\left({x}_{i1},{x}_{i2},\dots\:{x}_{in}\right)\:\:\:\:\:\:\:(i=\text{1,2},\dots\:,M)\:$$

4 $$\sum\limits_{{i = 1}}^{M} {\left[ {\hat{y}_{i} - y_{i} } \right]^{2} } \to min$$

Various levels of Kolmogorov-Gabor polynomials^90^ (Eq. 5) can be employed to establish a relationship between $x$ and $y$:

5 $$y = \alpha _{0} + \mathop \sum \limits_{{i = 1}}^{n} \alpha _{i} x_{i} + \mathop \sum \limits_{{i = 1}}^{n} \mathop \sum \limits_{{j = 1}}^{n} \alpha _{{ij}} x_{i} x_{j} + \mathop \sum \limits_{{i = 1}}^{n} \mathop \sum \limits_{{j = 1}}^{n} \mathop \sum \limits_{{k = 1}}^{n} \alpha _{{ijk}} x_{i} x_{j} x_{k} + \ldots$$

Previous research has indicated that utilizing the quadratic form of the Kolmogorov-Gabor polynomial (Eq. 6) achieves a favorable equilibrium between the intricacy and precision^90^. Also, the least-squares method is employed to ascertain the values of the model coefficients^91^.

6 $$y=G\left({x}_{i},{x}_{j}\right)={a}_{0}+{\alpha\:}_{1}{x}_{i}+{\alpha\:}_{2}{x}_{j}+{\alpha\:}_{3}{x}_{i}{x}_{j}+{\alpha\:}_{4}{x}_{i}^{2}+{\alpha\:}_{5}{x}_{j}^{2}$$

In this research, transformations are applied to the input variables, resulting in a notable enhancement in the efficiency of the developed models. These transformations encompass functions such as trigonometric, sigmoid, exponent, cube root, square root, and others. Taking into account the functions applied to the input variables, Eq. 6 can be reformulated as follows:

7 $$y={a}_{0}+{\alpha\:}_{1}f\left({x}_{i}\right)+{\alpha\:}_{2}g\left({x}_{j}\right)+{\alpha\:}_{3}f\left({x}_{i}\right)g\left({x}_{j}\right)+{\alpha\:}_{4}{f}^{2}\left({x}_{i}\right)+{\alpha\:}_{5}{g}^{2}\left({x}_{j}\right)$$

In Eq. (7), the transformation functions that can be applied to increase the accuracy of the ML modeling are displayed with $f\left(x\right)$ and $g\left(x\right)$.

### Models development

In order to effectively model using machine learning techniques, optimizing the model’s structure and hyper-parameters based on the problem’s features and datasets is necessary. The crucial structural parameters of the GMDH-NN encompass the layer count and the number of neurons. Quantitative optimization of neurons occurs through the self-organized procedure, where neurons that fail to meet the model’s accuracy criteria are eliminated from the model structure. However, achieving models that balance reasonable complexity and robustness necessitates restricting the number of layers. In order to achieve this objective, a set of trial and error analyses were conducted, leading to the implementation of a maximum limit of 10 layers for modeling all three TPPs.

The data inputted into the neural network is divided into two categories: a test set comprising 20% of the data and a training set comprising 80%. The initial category is dedicated to model evaluation, while the second category is used for training. Furthermore, 15% of training data points randomly are set aside as validation data to prevent over-fitting and assess the model’s generalizability.

It is essential to mention that the entire hybrid approach (ML modeling, MOO, and MCDM) has been implemented using MATLAB (R2021b) software.

### Evaluation criteria

Multiple statistical metrics are utilized to evaluate the effectiveness of GMDH-type ANN models, encompassing the following^92–94^:

8 $${\text{Coefficient}}\;{\text{ of}}\;{\text{ determination}}:\;R^{2} = 1 - \mathop \sum \limits_{{i = 1}}^{n} \frac{{\left( {Y_{{i,Pred}} - Y_{{i,Exp}} } \right)^{2} }}{{Y_{{i,Exp}}^{2} }}$$

9 $${\text{Correlation}}\;{\text{coefficient:}}\;R = \frac{{\mathop \sum \nolimits_{{i = 1}}^{n} \left( {Y_{{i,Exp}} - \overline{{Y_{{i,Exp}} }} } \right)\left( {Y_{{i,Pred}} - \overline{{Y_{{i,Pred}} }} } \right)}}{{\sqrt {\mathop \sum \nolimits_{{i = 1}}^{n} \left( {Y_{{i,Exp}} - \overline{{Y_{{i,Exp}} }} } \right)^{2} \mathop \sum \nolimits_{{i = 1}}^{n} \left( {Y_{{i,Pred}} - \overline{{Y_{{i,Pred}} }} } \right)^{2} } }}$$

10 $${\text{Mean}}\;{\text{absolute}}\;{\text{ percentage}}\;{\text{ error}}:\;MAPE\left( \% \right) = \frac{1}{n}\mathop \sum \limits_{{i = 1}}^{n} \left| {\frac{{Y_{{i,Pred}} - Y_{{i,Exp}} }}{{Y_{{i,Exp}} }}} \right| \times 100$$

11 $${\text{Mean}}\;{\text{squared}}\;{\text{error:}}\;MSE = \frac{1}{n}\mathop \sum \limits_{{i = 1}}^{n} \left( {Y_{{i,Exp}} - Y_{{i,Pred}} } \right)^{2}$$

${Y}_{i,Exp}$, ${Y}_{i,Pred}$, and $n$ show the actual values, output values, and the number of data, respectively. MAPE and MSE describe the error of the models, and R and R^2^ quantify the level of agreement between the observed data values and the predicted values, with values ranging from 0 to 1. Moreover, the deviation of predictions from data values can be visually represented using margin of deviation (MOD) diagrams by calculating relative error (RE). Each data point is individually evaluated using the following equation to determine the RE:

12 $${RE}_{i}\left(\%\right)=\frac{{Y}_{i,Exp}-{Y}_{i,Pred}}{{Y}_{i,Exp}}\times\:100$$

### Modeling results

Table 3 presents a comprehensive overview of various statistical metrics used to assess the performance of models in predicting thermophysical properties. The table reveals that these diverse criteria collectively validate the satisfactory precision of the constructed models. The models’ efficiency is attributed to three significant factors: the optimal selection of input transformations, the restriction of complexity, and the self-organizing process of the GMDH algorithm, which effectively selects the most suitable neurons. These three elements work together to ensure that the GMDH-based models achieve the desirable level of accuracy. During the testing phase for TC, the statistical criteria, including MSE, MAPE, R, and R^2^, exhibit values of 7.524E-05, 1.365, 0.9984, and 0.9968, respectively. This signifies the model’s capability to predict data points outside the training range accurately. The average relative error of less than 1.5% and the strong correlation between the model outputs and actual data validate the model’s effectiveness in predicting thermal conductivity. Like the TC, the dynamic viscosity predictive model demonstrates a valid modeling process. The statistical criteria, including MSE, MAPE, R, and R^2^, exhibit values of 0.227, 1.933%, 0.9985, and 0.9971, respectively. These values prove the reliability of the ANN-based developed model in predicting dynamic viscosity. Compared to the other two models, the predictive model for specific heat capacity outperforms them in accurately matching the actual data with the model outputs during the testing stage. This is evident from the high correlation coefficients, with R and R^2^ values of 0.9987 and 0.9973, respectively. Furthermore, the SHC predictive model exhibits an average relative error of less than 1%.

The assessment of data-driven models is heavily contingent on the pivotal benchmark of the testing phase. Nonetheless, assessing the model’s accuracy on the training data can provide valuable insights into the training process. Based on the observations from Table 3, it is evident that all three models exhibit a noteworthy level of consistency between the accuracy achieved during the training phase and the results obtained during the testing phase. This consistency indicates the satisfactory performance of the validation process in modeling. Specifically, the MAPE and R criteria exhibit values of 1.783% and 0.9971 for the TC predictive model during the training stage. Similarly, the DV predictive model demonstrates values of 1.539% and 0.9991 for MAPE and R, respectively. The SHC predictive model also showcases high accuracy, with values of 0.599% and 0.9987 for criteria MAPE and R, respectively.

The visual representation of the structure and sub-models for each predictive model are presented in the appendix section.

**Table 3:** Statistical criteria of the ANN-based models.

TPP	Dataset	MSE	MAPE (%)	*R*	*R* ^2^
TC	Testing	7.524E-05	1.365	0.9984	0.9968
	Training	1.286E-04	1.783	0.9971	0.9942
DV	Testing	0.227	1.933	0.9985	0.9971
	Training	0.133	1.539	0.9991	0.9982
SHC	Testing	3.533E-04	0.684	0.9987	0.9973
	Training	3.405E-04	0.599	0.9987	0.9975

**Fig. 5:**
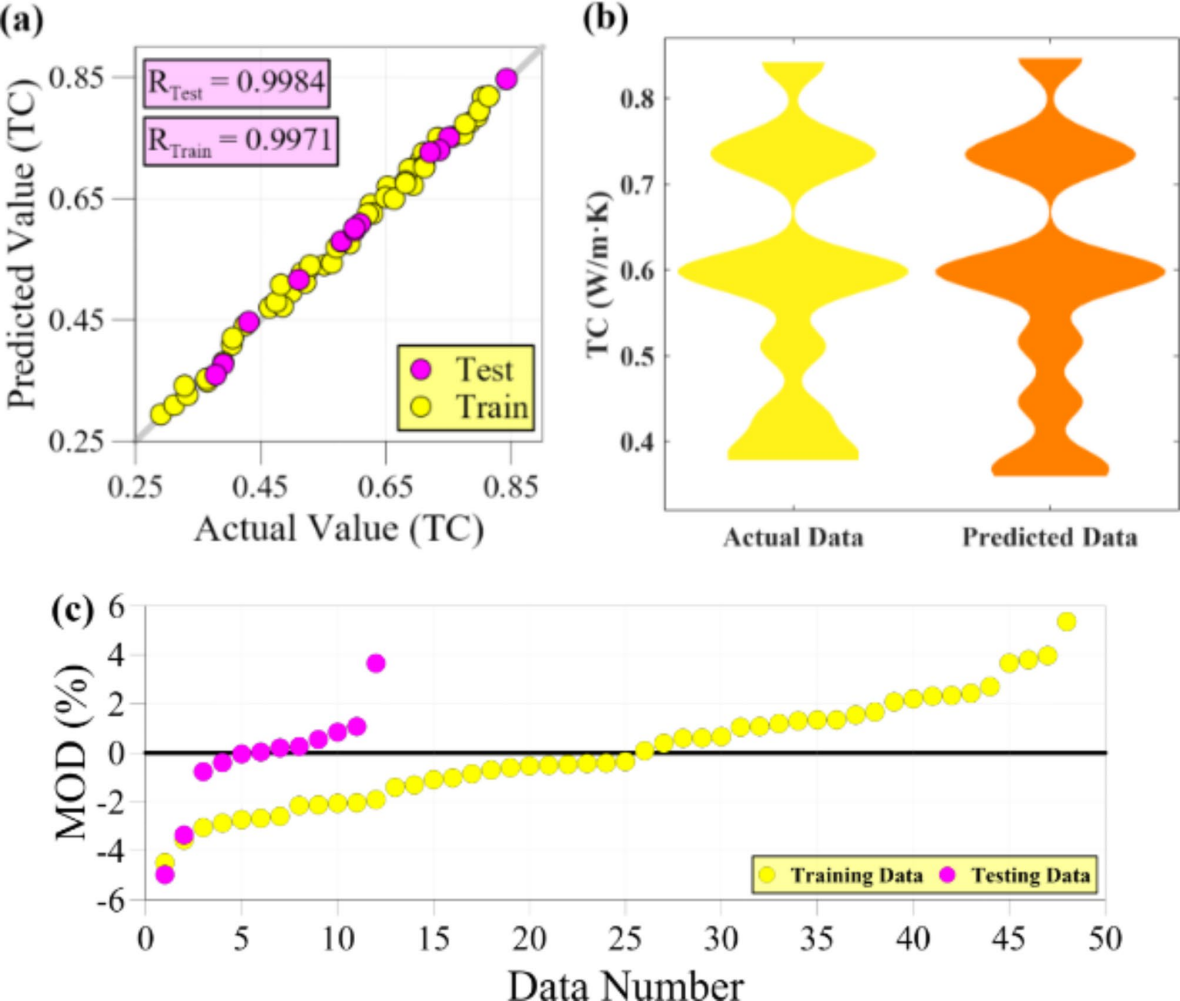
Accuracy evaluation of ANN-based developed model for TC, (a) Regression graph, (b) Violin plots, and (c) MOD chart.

**Fig. 6:**
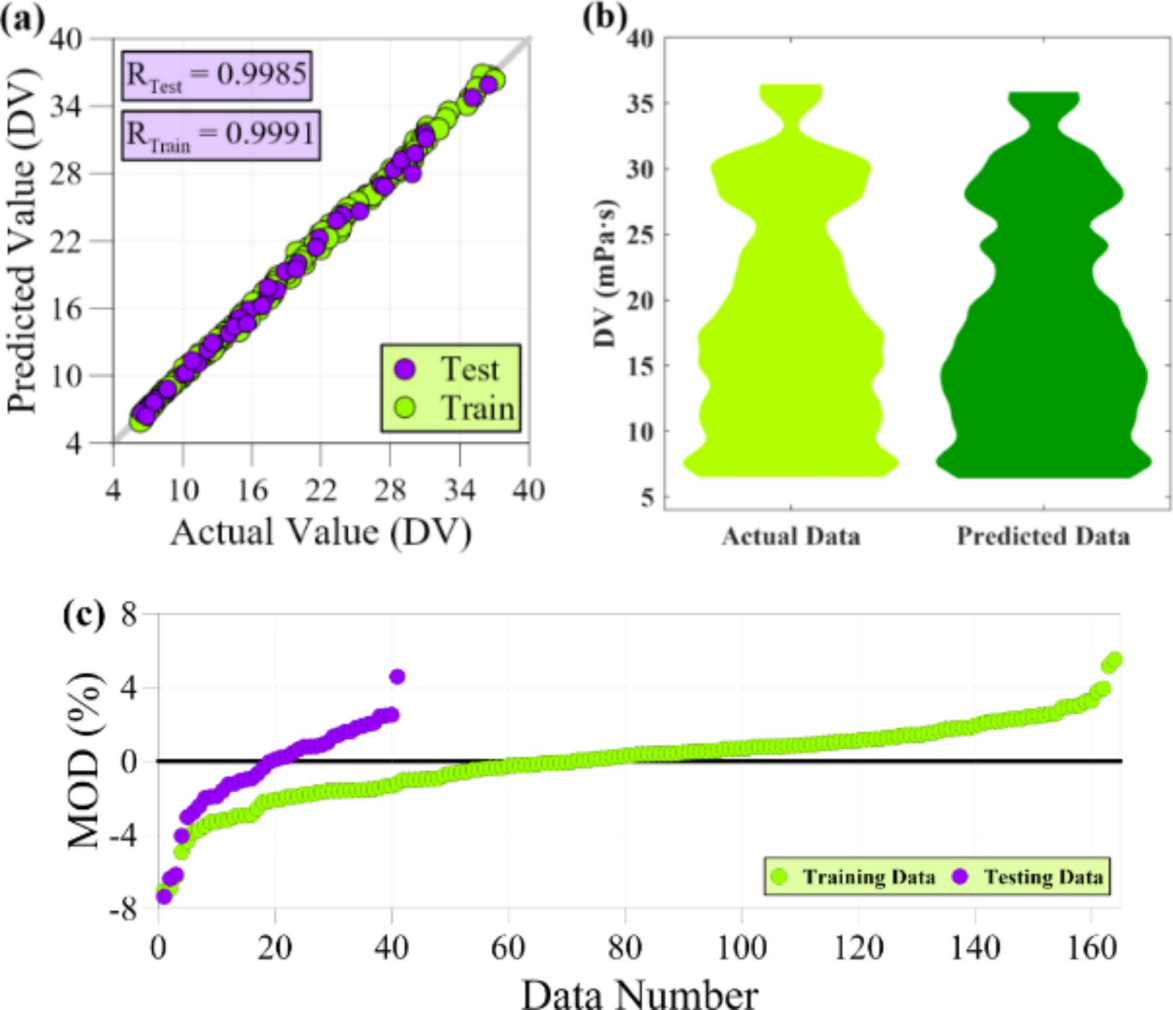
Accuracy evaluation of ANN-based developed model for DV, (**a**) Regression graph, (**b**) Violin plots, and (**c**) MOD chart.

**Fig. 7:**
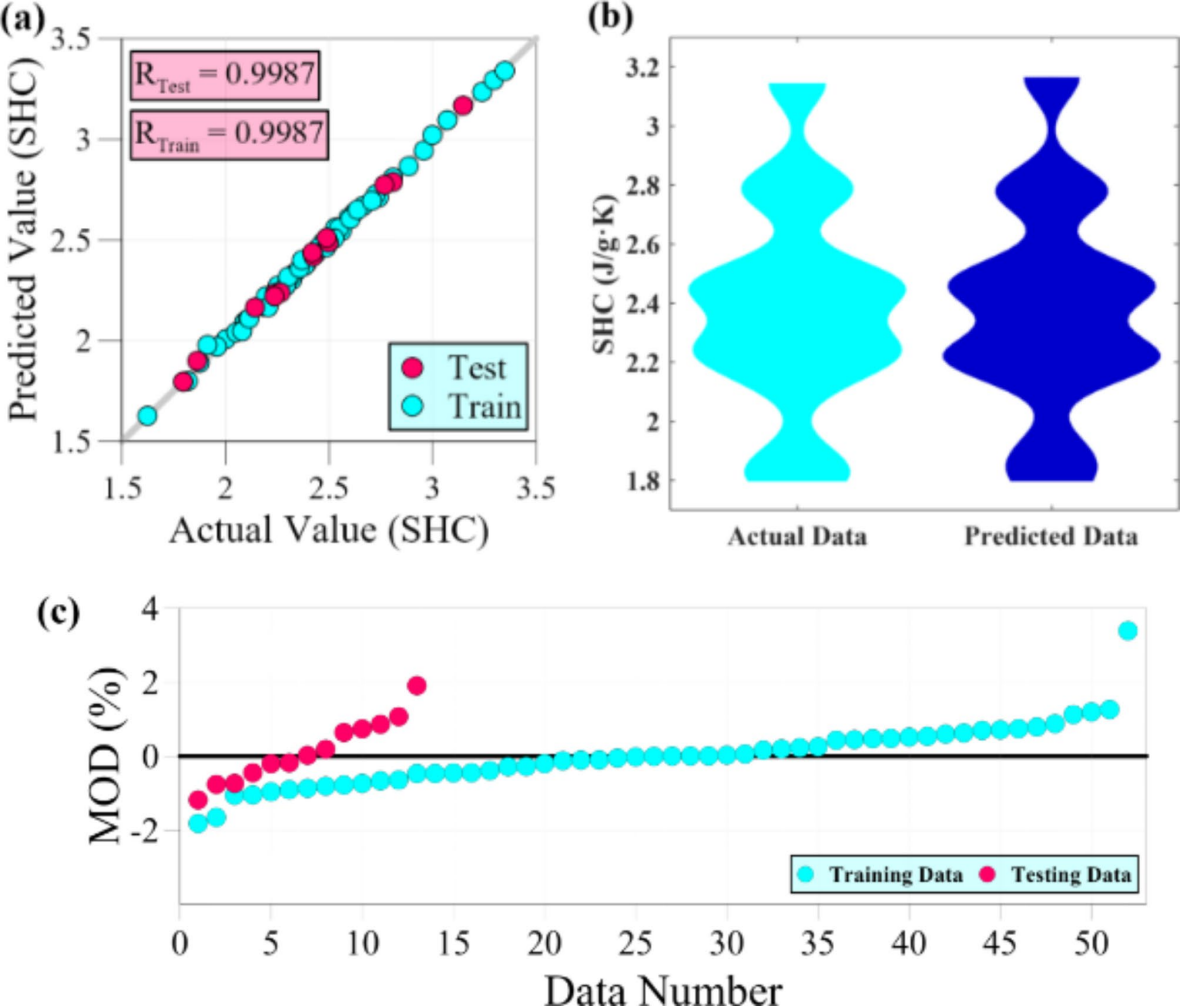
Accuracy evaluation of ANN-based developed model for SHC, (**a**) Regression graph, (**b**) Violin plots, and (**c**) MOD chart.

The validity of the developed models for predicting TC, DV, and SHC can be better understood by referring to Figs. 5 and 6, and 7, respectively. These figures provide a visual comparison of the error and fit of the data points, offering valuable insights into the performance of the models. Figure 5 presents a regression graph, violin plot, and MOD chart of the thermal conductivity predictive model during the testing and training phases. These visual representations enable a comprehensive analysis of the model’s performance and ability to predict TC accurately. Figure 5 (a), in conjunction with Table 3, confirms the findings related to the precision of the predictive model for TC. As the figure shows, many data points align closely with the Y = X line, revealing a strong agreement between the predicted and actual values. However, it is worth noting that a few testing and training data points deviate slightly from the Y = X line, resulting in a small error for the model. Figure 5 (b) compares the probability density function (PDF) of the modeled outputs and the experimental values for the testing data, presented as a violin plot. The figure reveals that for values above 0.55 W/m·K, there is a notable agreement between the output PDF and the actual values. This suggests the model performs well in accurately predicting data within this range. However, for values below 0.55 W/m·K, a slight difference is observed, indicating a higher probability of error in predicting data within this specific range. Figure 5 (c) illustrates the relative deviation of the model’s outputs from the target values for each data point, depicted as a MOD graph. As the figure displays, the relative error for the testing data falls within the interval of -4.97% < MOD < 3.65%. Similarly, for the training data, the relative error is situated within the − 4.49% < MOD < 5.36% range. Based on the observations from the figure, it is evident that only two data points in the training data exhibit an absolute relative error of more than 4%. On the other hand, three specific data points stand out for the testing data with relative error of -4.97%, -3.36%, and 3.65%. These data points impose higher errors on the predictive model compared to the other data points in the testing phase. Aside from the few data points with significant relative errors, most test data points are scattered close to the zero error line. This observation suggests that, in general, the developed model achieves a good balance between overestimating and underestimating the actual data.

Figure 6 visually analyzes the model’s performance in predicting dynamic viscosity. It includes a regression graph, violin plot, and MOD chart for testing and training data. In Fig. 6 (a), it is evident that a significant number of data points are located on the Y = X line, demonstrating a strong agreement between the outputs and actual values of dynamic viscosity. This compliance suggests that the model’s predictions are generally accurate and aligned with the observed data. However, a few testing and training data points deviate slightly from the Y = X line. These deviations indicate that the model may not predict these specific data points perfectly. Figure 6 (b) compares the modeled outputs’ PDF with the testing data’s experimental PDF. Upon analysis of the figure, it becomes apparent that scattered differences are observed in specific values. However, despite these discrepancies, the two violin charts have an overall high level of compatibility. Figure 6 (c) presents the relative deviation of the model’s outputs from the target values for each data point, as depicted in the MOD chart. According to the figure, the relative error for the testing data falls within the interval of -7.36% < MOD < 4.58%. Similarly, the training data’s relative error is within the range of -7.03% < MOD < 5.50%. Upon closer examination, it is observed that there are seven data points in the training data that exhibit an absolute relative error of more than 4%. These data points indicate instances where the model’s predictions deviate significantly from the actual values of dynamic viscosity in the training set. Similarly, five specific data points stand out for the testing data with relative errors of 4.58%, -4.06%, -6.17%, -6.38%, and − 7.36%. These specific data points with more significant relative errors impose higher errors on the predictive model than other data points. However, aside from these few data points, most test data points are scattered close to the zero error line. Analyzing the data error distribution in Fig. 6 (c), it is evident that there is an acceptable balance between overestimation and underestimation of the experimental values in the training phase. Nevertheless, it is worth noting that there are more data points in the testing phase with negative relative error, indicating that the model tends to overestimate the actual values of dynamic viscosity.

Figure 7 provides a comprehensive visual analysis of the model’s performance in predicting specific heat capacity. In Fig. 7 (a), it is clearly observed that a substantial number of data points lie along the Y = X line. This alignment indicates a strong agreement between the predicted and actual values of SHC. Figure 7 (b) compares the PDF of the modeled outputs and the PDF of actual values. Upon careful examination of the figure, it becomes evident that slight differences are observed in certain areas. However, despite these discrepancies, there is a robust overall resemblance between the two violin charts, indicating a high level of compatibility. Figure 7 (c) illustrates the MOD plot for testing and training datasets. Based on the analysis of the figure, the relative error for the testing data is found to be within the range of -1.19–1.90%. Similarly, the relative error falls within the interval of -1.82–3.39% for the training data. Upon closer inspection, it is observed that eight data points in the training phase exhibit an absolute relative error of more than 1%. However, only one data point shows a significant error of 3.39%, while the remaining training data follows a consistent error trend. Likewise, in the case of the testing data, three distinct data points stand out with relative errors of -1.19%, 1.07%, and 1.90%. Apart from these few data points, the rest of the testing data points exhibit a relative error of less than 1%. After excluding the single data point with a high relative error (3.39%), the error distribution depicted in Fig. 7 (c) indicates a satisfactory balance between overestimation and underestimation of the experimental values during the testing and training phases.

## Multi objective optimization

Maximizing the TC and SHC of nanofluids while simultaneously minimizing their DV is a critical challenge in practical PV/T systems. Given the interplay between operational, material, and flow-related factors, achieving a single optimal solution for all objectives is often impractical. To tackle this multi-objective optimization problem, the Pareto front approach provides a comprehensive framework. By exploring the solution space, this approach identifies a set of optimal trade-offs, rather than a single best solution. This is particularly useful when conflicting objectives make it difficult to achieve a single optimal outcome. To identify these Pareto optimal solutions, this study employs the well-established MOPSO algorithm, alongside a novel approach, the MOTEO algorithm. The three-objective optimization problem addressed in this research can be mathematically formulated as follows:

13 $$\left\{ {\begin{array}{*{20}c} {Maximize~TC = f\left( {MF,T} \right)~~~~~~~~~~~~~~~~~} \\ {Minimize~DV = g\left( {MF,T} \right)~~~~~~~~~~~~~~~~~} \\ {Maximize~SHC = h\left( {MF,T} \right)~~~~~~~~~~~~~~} \\ {Subject~to~\left\{ {\begin{array}{*{20}c} {0 \le MF \le 0.4~\left( {wt\% } \right)} \\ {25 \le T \le 75~\left( {^\circ C} \right)~~~~~~~} \\ \end{array} } \right.~} \\ \end{array} } \right.$$

The optimization process employs the GMDH neural network models as $f$, $g$, and $h$ functions. It is essential to highlight that the temperature data associated with each objective exhibit distinct ranges. The common temperature range of targets, i.e., 25 to 75 °C, is used in the current multi-objective optimization.

### Multi-objective thermal exchange optimization (MOTEO)

The MOTEO algorithm is grounded in the principles of thermal exchange optimization (TEO), drawing inspiration from the physical phenomenon of Newton’s law of cooling^95^. This methodology conceptualizes search agents as cooling bodies, where their temperature values correspond to the optimization parameters. TEO partitions search agents into two groups: the ambient medium and cooling entities. Initially, cooling entities are randomly dispersed throughout the solution space and evaluated using cost functions. A thermal memory (TM) system retains optimal solutions, safeguarding promising regions of the search space and intensifying their exploration. TEO uses the fundamental principle of Newton’s law of cooling, which states that the rate at which a body’s temperature decreases is directly proportional to the temperature difference between the body and its environment. This principle governs the cooling process within the TEO algorithm. This cooling principle is applied to adjust the temperatures of search agents, thereby facilitating both a comprehensive exploration of the solution space and convergence towards optimal solutions^95^. MOTEO, a multi-objective variant of TEO, incorporates a distinctive ranking strategy. By employing non-dominated sorting, it organizes solutions into distinct Pareto fronts, adeptly handling multiple objectives. The MOTEO algorithm employs a series of key stages, adapted from its single-objective predecessor. The following outline details the sequential execution of these steps within the MOTEO algorithm^95^:

1. Temperature Initialization: Initially, search agents are randomly assigned temperatures across the solution space. This random initialization ensures a diverse range of potential solutions, with temperatures representing the optimization variables.
2. Solution Evaluation: Each search agent, or cooling element, is evaluated against the defined objective functions. This evaluation process quantifies the performance of each solution in relation to the optimization objectives.
3. Pareto Front Ranking: A non-dominated sorting algorithm is used to classify solutions into Pareto fronts. Solutions that are not dominated by any other solution are assigned a lower rank. This ranking process categorizes solutions into distinct fronts, aiding in the identification of optimal solutions.
4. Crowding Distance Assignment: Subsequent to non-dominated sorting, a crowding distance metric is calculated for each solution within the same Pareto front. This metric encourages diversity among solutions by prioritizing those located in less populated regions of the objective space.
5. Solution Selection: The selection mechanism uses both rank and crowding distance to identify the most promising solutions for the next generation. Solutions with lower domination ranks and higher crowding distances are prioritized, ensuring a balance between converging towards optimal solutions and maintaining diversity within the population.
6. Temperature Adjustment: The temperatures of the cooling agents are updated based on Newton’s law of cooling. This temperature adjustment is crucial for exploring the solution space comprehensively and avoiding premature convergence to suboptimal solutions. The new temperature is calculated by multiplying the previous temperature by a decay factor.
7. Local Optima Avoidance: To prevent the algorithm from becoming trapped in suboptimal solutions, a technique is employed that randomly modifies one dimension of a randomly selected solution within its feasible range. This perturbation helps to explore new regions of the solution space.
8. Termination Criteria: The algorithm iteratively evaluates, sorts, and updates solutions until a predetermined maximum number of iterations is reached. Upon termination, the identified optimal solutions are recorded.

Table 4 presents the parameter settings for the MOTEO algorithm used in the current multi-objective optimization. The values provided were determined through a series of trial-and-error adjustments to achieve optimal compatibility between the algorithm and the problem requirements.

**Table 4:** Parameter setting of the MOTEO algorithm.

MOTEO Parameters	Value
Population size	200
Iterations	500
Personal learning coefficient	1.1
Global learning coefficient	2.2
Mutation probability	0.4

### Multi-objective particle swarm optimization (MOPSO)

MOPSO is a well-known meta-heuristic algorithm employed to tackle multi-objective optimization problems. It draws inspiration from the collective behavior observed in nature, such as fish schooling or bird flocking^96^. By utilizing a swarm of particles, MOPSO explores the problem space, with each particle representing a potential solution. These particles adjust their positions and velocities by considering personal and global best solutions. Through iterative updates, MOPSO aims to discover solutions along the Pareto front, which captures the trade-off between conflicting objectives. MOPSO’s ability to strike a balance between exploitation and exploration has made it a widely adopted approach for seeking diverse optimal solutions. The procedure for MOPSO can be summarized in the following sequence^96^:

1. Initialization: The population of particles is randomly distributed within the search space. Each particle is a potential solution for the optimization problem and possesses its position and velocity.
2. Evaluation: The fitness of each particle is determined by evaluating the objective function values corresponding to its current position.
3. Personal best update: For each particle, update its personal best position and fitness value, representing the most optimal solution encountered by the particle thus far.
4. Global best update: By comparing all the personal best positions, identify the best solution and update the global best position accordingly. This global best position represents the overall best solution discovered by the entire swarm.
5. Velocity update: The velocity of each particle is adjusted by considering its current velocity, personal best position, and global best position. This adjustment determines both the direction and magnitude of movement for each particle, influencing their exploration and exploitation capabilities within the search space.
6. Position update: Based on their current position and the updated velocity, the position of each particle is modified. This adjustment enables the particles to move towards potentially improved solutions within the search space, promoting exploration and exploitation of the problem domain in the quest for optimal solutions.
7. Termination criterion: The termination criterion is assessed to determine if it has been met. This criterion can take various forms, such as reaching a maximum number of iterations, attaining a desired fitness value, or surpassing a predefined threshold for improvement.
8. The steps 2 to 7 are iteratively repeated until the termination criterion is fulfilled.

The high validity and reliability of MOPSO have resulted in its widespread application for optimizing thermo-fluid problems^97–100^. Table 5 outlines the configuration for the MOPSO algorithm applied in this multi-objective optimization. These values were derived from an iterative trial-and-error process to ensure the algorithm’s effective alignment with the problem’s specific requirements.

**Table 5:** Parameter setting of the MOPSO algorithm.

MOPSO Parameters	Value
Swarm size	200
Repository size	200
Iterations	500
Inertia weight	0.6
Individual confidence factor	2
Swarm confidence factor	2
Number of grids in each dimension	20
Uniform mutation percentage	0.5
Maximum velocity (%)	10

### Pareto optimal points

**Fig. 8:**
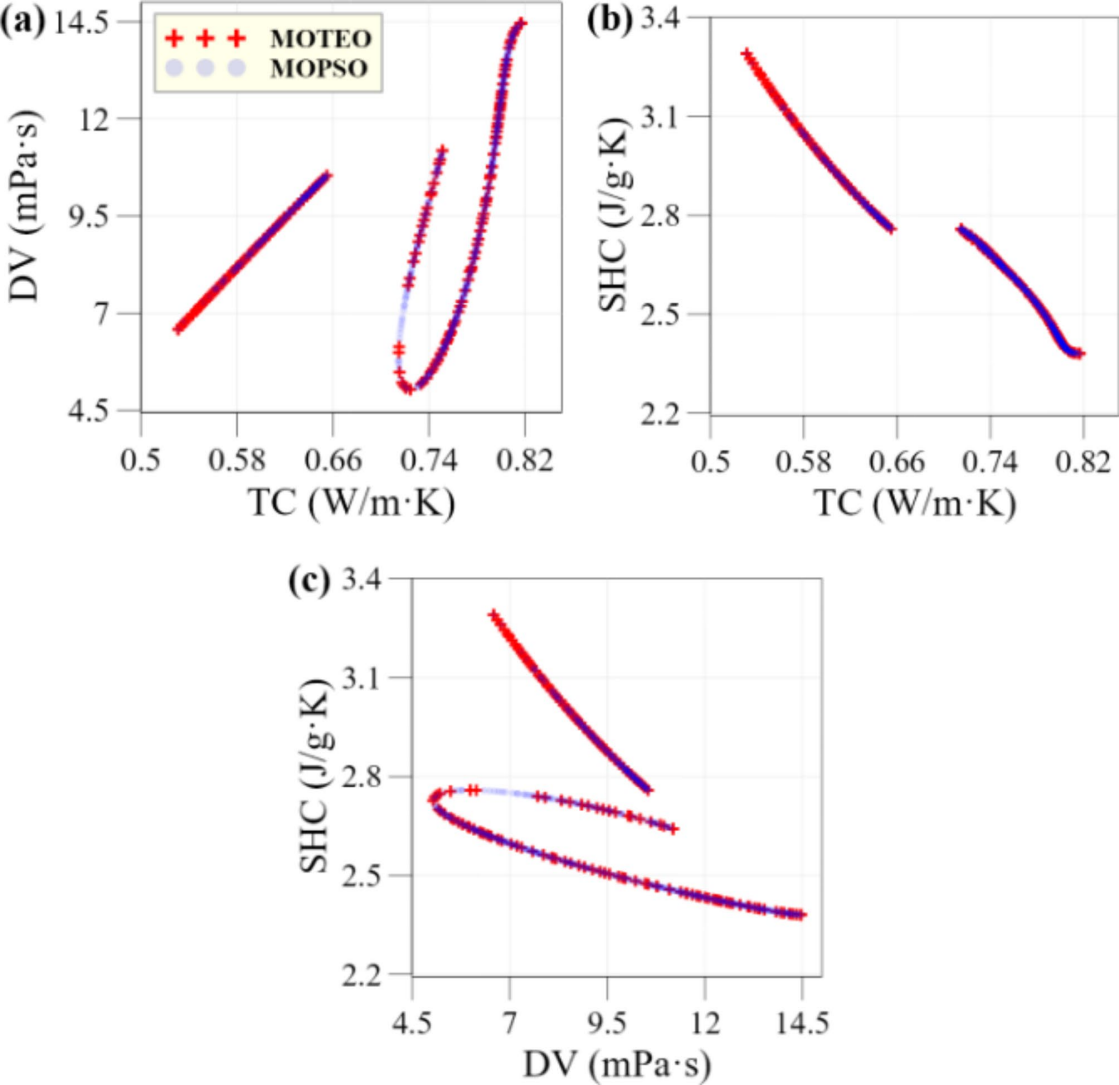
Pareto optimal points resulting from MOPSO and MOTEO algorithms.

Figure 8 shows the optimal Pareto points obtained using the MOPSO and MOTEO algorithms. As illustrated, both algorithms produce similar Pareto fronts across all three subplots, with minor differences in coverage. MOPSO offers a slightly broader distribution of optimal points, which may provide greater flexibility in selecting conditions based on the desired balance of TPPs. This suggests that, while both algorithms are effective for this multi-objective optimization task, MOPSO may have a slight advantage in solution diversity, which could be beneficial for scenario-based design in solar PV/T applications.

**Fig. 9:**
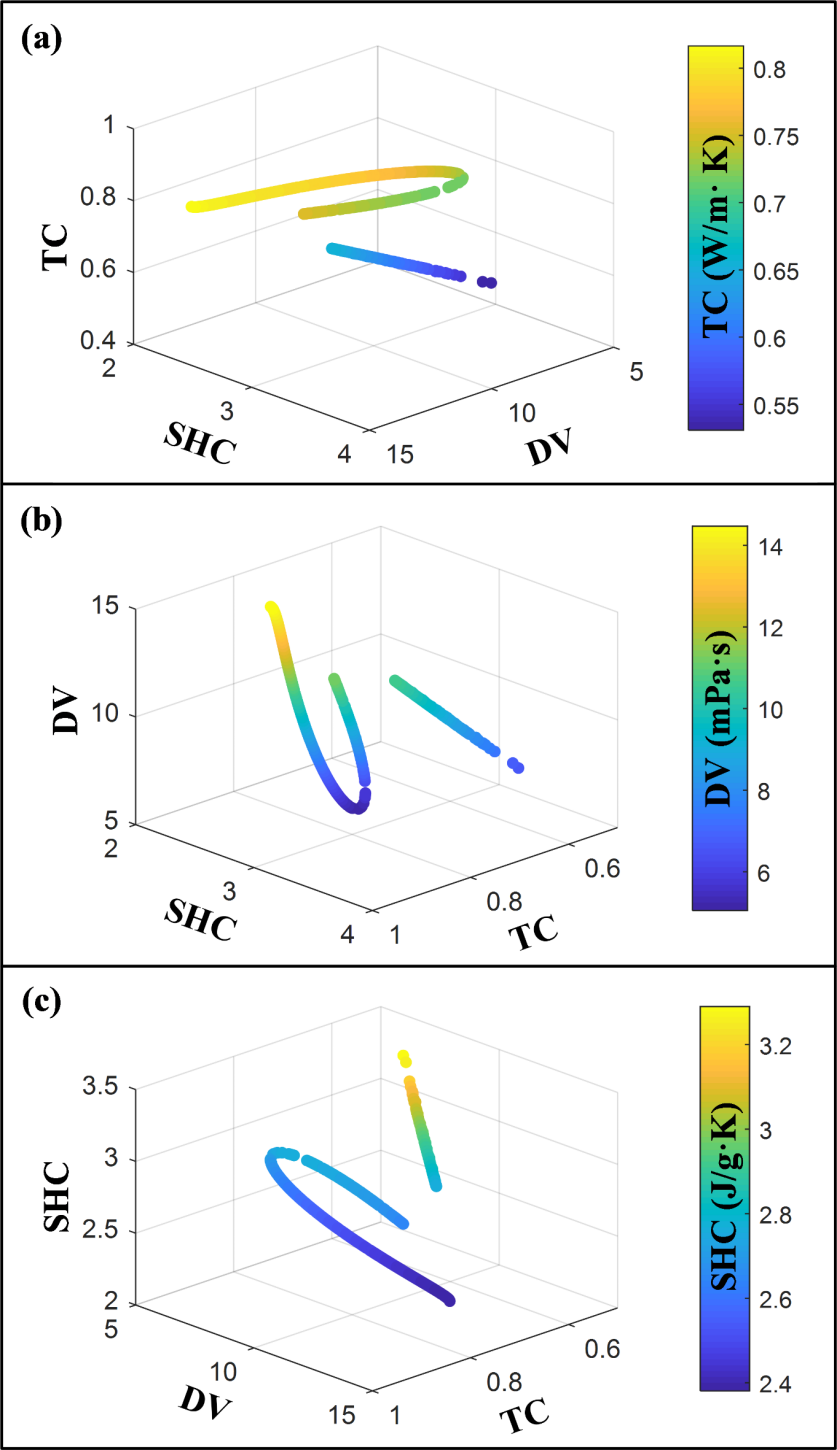
Three-dimensional visualization of the Pareto front generated by the MOPSO algorithm.

To emphasize the Pareto front obtained from the MOPSO algorithm, Fig. 9 presents three-dimensional visualizations focused on each objective. The 3D plots in Fig. 9 demonstrate the optimization approach’s effectiveness across three key metrics: convergence, coverage, and diversity. The Pareto front solutions consistently align near the optimal front, indicating strong convergence toward optimal values. The solutions span a broad range across all objectives, ensuring thorough exploration of the objective space and providing a variety of trade-off options. Additionally, the well-distributed placement of solutions reflects diversity, offering flexibility for selecting solutions that balance priorities among TC, DV, and SHC based on specific application requirements.

**Fig. 10:**
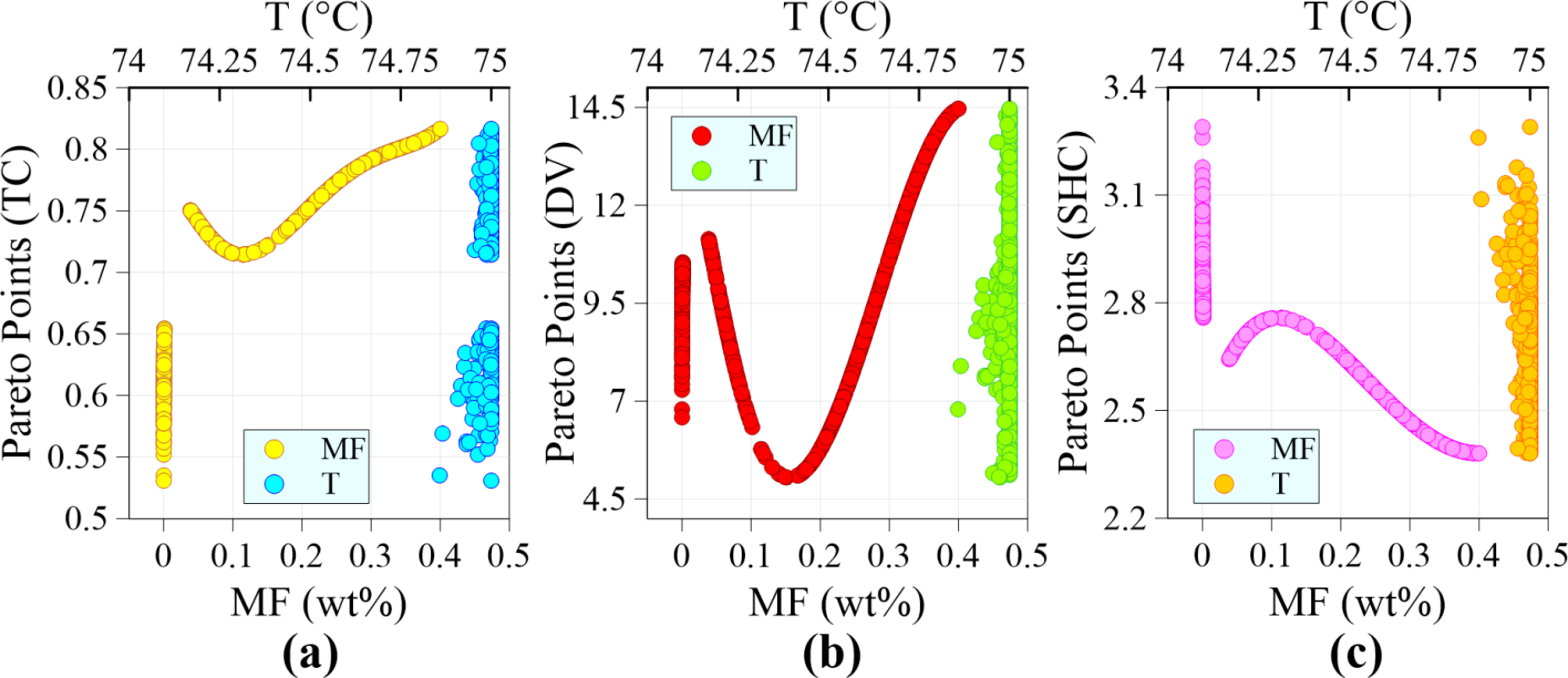
Optimal values of (**a**) TC, (**b**) DV, and (**c**) SHC, and their corresponding input variables.

Figure 10 illustrates the variations in the optimal values of the input variables with respect to different objectives, based on the Pareto points obtained from the MOPSO algorithm. As observed, the optimal points exhibit a diverse range of MFs. However, there is a specific range, approximately between 0.001% and 0.039%, where no optimal point is found. This observation suggests that for MXene-based nanofluids to have the potential to achieve an optimal state when used in IL/DEG fluid, the MXene mass fraction value must exceed a certain minimum threshold. The base fluid, characterized by an MF = 0, accounts for approximately 27.8% of the optimal points. The presence of the base fluid in the optimal points leads to a specific pattern where the optimal thermal conductivity values tend to be relatively low, ranging from 0.53 to 0.65. Furthermore, these optimal points also exhibit optimal dynamic viscosity values that tend to fall within the intermediate range of 6.5 to 10.5. Additionally, the optimal specific heat capacity values are relatively high, ranging from approximately 2.75 to 3.3. These observations suggest that in scenarios where the relative significance of heat capacity outweighs the other two objectives, the base fluid can be considered the optimal state. In the remaining optimal points influenced by the inclusion of MXene nanoparticles, with an increase of MF, a similar pattern is observed in the Pareto front for TC and DV. Initially, the Pareto front has a downward slope, indicating a reduction in their optimal values. However, beyond a certain threshold, the Pareto front has an upward slope, suggesting an improvement in TC and DV values with a further increase in the mass fraction of MXene nanoparticles. The trend is the opposite for SHC. As the MF of MXene nanoparticles increases, the Pareto front for SHC initially increases, indicating an improvement in its optimal values. However, the Pareto front has a downward slope beyond a certain point, suggesting a decline in SHC values with a higher MF of MXene nanoparticles.

Notably, a substantial portion, precisely 42.2%, of the optimal points corresponds to a mass fraction of MXene nanoparticles less than 0.1%. This implies that many optimization scenarios for nanofluids involve relatively low concentrations of MXene nanoparticles. Interestingly, only a tiny proportion, specifically 6.6%, of the optimal states fall within the concentration range of 0.1–0.2%. In contrast, a relatively more significant percentage of optimal points, accounting for 26.2% and 25%, are associated with MF ranges of 0.2–0.3% and 0.3–0.4%, respectively. It can be observed that more than 50% of the optimal states are concentrated in the upper half of the MF range (0.2–0.4%). In contrast, the temperature of the optimal points is confined to a narrow range around its maximum value (75 °C). This limitation is primarily due to the simultaneous enhancement of all three objectives as the temperature increases. The statistical analysis of the Pareto front reveals that a relatively small percentage (16.4%) of the optimal points have temperature values below 75 °C. Additionally, it is noteworthy that the minimum optimal temperature reported is 74.86 °C, which is very close to the maximum value. It is important to note that this research presents optimal points by evaluating key TPPs of MXene nanofluids from a multi-objective perspective. Therefore, the optimal values for nanofluid temperature is determined solely based on the nanofluid’s performance in a PV/T hybrid solar system. The optimal operating temperature for these systems can vary depending on the impact of PV and thermal components. Evaluating and optimizing operating temperature involves considering the type of system and details such as solar irradiance, optical concentration, load resistance, ambient temperature, hybrid cooling strategies, thermal effects on PV cells and waste heat recovery details, etc.

## Multi-criteria decision-making (MCDM)

Selecting the optimal points from the multitude of solutions delivered by the Pareto front can be daunting for designers. In order to tackle this challenge, multi-objective decision-making methods prove to be valuable tools. This study employs the TOPSIS approach as one of the well-established decision-making tools. The TOPSIS method employs a sum function, where the proximity to the ideal point is used as the basis for selecting the desirable points. Previous studies across various disciplines have highlighted the effectiveness of the TOPSIS method^101,102^.

### TOPSIS approach

TOPSIS helps decision-makers evaluate and rank alternatives based on their performance across multiple criteria. The procedure that the decision maker follows utilizing TOPSIS can be summarized as follows:

1. Create a matrix with alternatives as rows and criteria as columns, containing numerical values representing performance.
2. Normalize the matrix to eliminate measurement scale biases.
3. Assign weights to each criterion and multiply the normalized values by their corresponding weights.
4. Identify the best and worst performance values for each criterion.
5. Measure the similarity of each alternative to the ideal and negative-ideal solutions using Euclidean distances.
6. Calculate the relative closeness of each alternative based on the distances to the ideal and negative-ideal solutions.
7. Rank the alternatives based on their relative closeness values, with the highest value indicating the most preferred solution.

### Selection of desirable points

The Pareto front refers to a collection of optimal solutions that are not ranked higher or lower than one another but outperform other solutions within the given problem space. Transitioning from one solution to another within the Pareto front does not result in simultaneous improvement of all objectives. Instead, it involves enhancing one objective while sacrificing or compromising another objective. Various solutions within the Pareto front highlight the varying significance or priority assigned to the objectives. This research utilizes TOPSIS and a scenario-based approach to address real-world decision-making by assigning varying importance factors (weights) to objectives. Weighting in MCDM reflects design complexities, enabling prioritization based on each objective’s significance. This method aids in identifying optimal alternatives that align with the decision-maker’s preferences, resulting in tailored, practical solutions. By evaluating different scenarios (as shown in Table 6), the approach allows for flexible analysis, adapting to specific project needs and external constraints. This flexibility ensures a realistic alignment with real-world priorities, facilitating shifts in importance based on project objectives and practical conditions.

**Table 6:** The proposed optimal points through TOPSIS process for different scenarios.

Points	Importance Factor (Weight)			Input variables		Objectives		
	TC	DV	SHC	T (°C)	MF (wt%)	TC (W/m·K)	DV (mPa·s)	SHC (J/g·K)
**A**	1	1	1	74.97	0.142	0.719	5.124	2.742
**B**	3	1	1	75.00	0.235	0.766	7.177	2.589
**C**	1	3	1	74.97	0.149	0.721	5.063	2.734
**D**	1	1	3	75.00	0.000	0.531	6.584	3.290
**E**	2	3	1	75.00	0.170	0.731	5.112	2.708
**F**	3	2	1	75.00	0.201	0.747	5.814	2.654
**G**	3	1	3	74.99	0.130	0.716	5.311	2.751

Table 6 displays the desirable points acquired through the TOPSIS method for various scenarios. Different design scenarios are established by considering the importance factors assigned to the objectives. It is important to note that, for each scenario (points A to G), only the top-ranked solution (rank 1) determined by the TOPSIS method is presented in Table 6. From a general observation of the table, it is noticeable that the optimal temperature tends to be near the maximum value. Furthermore, the table demonstrates wide ranges of optimal MF, corresponding with the findings in Fig. 10. Point A represents a scenario where all three objectives hold equal importance. In this particular situation, the design requirements can be met by achieving a mass fraction of 0.142%. As the importance of thermal conductivity increases in point B, a higher concentration of MXene is necessary compared to Point A. This indicates that a greater emphasis on thermal conductivity necessitates a higher mass fraction of MXene to meet the design objectives. In contrast, when the importance of viscosity is increased in point C, the mass fraction is expected to approach zero (base fluid). However, contrary to expectations, the mass fraction does not tend to reach zero. This observation aligns with previous research conducted by Mao et al.^54^ and Bao et al.^55^, which concluded that MXene nanoparticles do not significantly change dynamic viscosity.

At point D, where the importance of specific heat capacity surpasses that of the other two objectives, it is observed that the best performance is achieved with the base fluid (MF = 0). Points E and F represent scenarios where the SHC is considered the least important, while the importance of TC and DV varies. In these scenarios, it can be observed that a greater mass fraction is required when the importance of thermal conductivity is higher. On the other hand, when the importance of dynamic viscosity is high, it is observed that a lesser mass fraction is needed. Point G represents conditions where TC and SHC are considered the most important, often in solar applications. In this particular scenario, it is observed that an MF of 0.13% can provide favorable conditions to meet the design requirements.

## Conclusion

The optimization of thermophysical properties in nanofluids has proven to be instrumental in enhancing the efficiency of thermal systems. In this research, a hybrid approach was proposed to optimize the properties of Mxene-based nanofluids under various scenarios. In this study, the input variables were the MXene mass fraction and temperature. The study’s primary objective was to optimize the nanofluid TPPs by maximizing the thermal conductivity and specific heat capacity while minimizing the dynamic viscosity. These three parameters are crucial in characterizing nanofluids and play a vital role in enhancing the performance of PV/T solar systems. In the current hybrid approach, the powerful GMDH-type ANN technique was used to model target variables in terms of input variables. The obtained models were integrated into the MOPSO and MOTEO algorithms, forming a three-objective optimization problem. In the final stage, the TOPSIS technique was employed to identify the desirable optimal points from the Pareto front obtained through the MOSPO algorithm. The approach proposed in this research offers valuable insights into identifying the optimal conditions for MXene-based nanofluids used in solar systems, considering real-world scenarios. The main findings of the study can be summarized as follows:

- The GMDH-NN under structural optimization demonstrated outstanding performance in predicting the objectives of the problem.
- The developed models for TC, DV, and SHC demonstrated a strong performance on the testing data by recording the correlation coefficient of 0.9984, 0.9985, and 0.9987, respectively. The mean absolute percentage error of the models was reported as 1.365%, 1.933%, and 0.684%, respectively.
- The outcomes of the multi-objective optimization revealed that the Pareto points exhibited a broad range of MXene mass fractions. However, the temperature of these optimal points was found to be constrained within a narrow range near the maximum value.
- Approximately 27.8% of the Pareto optimal points had an MF = 0, indicating the base fluid without any MXene additive. In these optimal points, the specific heat capacity exhibited notably high values.
- Over 50% of the optimal states were concentrated in the MXene mass fraction’s upper half (0.2–0.4%). In these optimal states, the TC of the nanofluids could reach high values.
- Assigning weights or importance factors to objectives in the multi-criteria decision-making process offers the opportunity to incorporate real-world design scenarios.
- In scenarios where TC precedes other objectives, the TOPSIS method recommends utilizing an MXene mass fraction of over 0.2%. Alternatively, if dynamic viscosity holds greater importance, decision-makers can opt for an MXene mass fraction ranging from 0.15 to 0.17%. Also, when heat capacity becomes the primary concern, utilizing the base fluid without any MXene additive is advisable.

In future research, exploring the challenges posed by inflexible factors like temperature variations and MXene mass fraction fluctuations in real-world applications could lead to investigating the constrained multi-objective optimization problem (CMOP).

## Electronic supplementary material

Below is the link to the electronic supplementary material.

Supplementary Material 1

## Data Availability

The datasets used and analyzed during the current study available from the corresponding author on reasonable request.
