# Correction: Integrating artificial neural networks, multi-objective metaheuristic optimization, and multi-criteria decision-making for improving MXene-based ionanofluids applicable in PV/T solar systems

**DOI:** 10.1038/s41598-025-99770-7

**Published:** 2025-05-13

**Authors:** Tao Hai, Ali Basem, As’ad Alizadeh, Kamal Sharma, Dheyaa J. jasim, Husam Rajab, Abdelkader Mabrouk, Lioua Kolsi, Wajdi Rajhi, Hamid Maleki, Narinderjit Singh Sawaran Singh

**Affiliations:** 1 Artificial Intelligence Research Center (AIRC), College of Engineering and Information Technology, Ajman University, P.O. Box: 346, Ajman, UAE; 2 Faculty of Data Science and Information Technology, INTI International University, 71800 Nilai, Malaysia; 3 Faculty of Engineering, Warith Al-Anbiyaa University, Karbala, 56001 Iraq; 4 Department of Civil Engineering, College of Engineering, Cihan University-Erbil, Erbil, Iraq; 5 Institute of Engineering and Technology, GLA University, Mathura, U.P 281406 India; 6 Department of Petroleum Engineering, Al-Amarah University College, Maysan, Iraq; 7 College of Engineering, Department of Mechanical Engineering, Najran University, King Abdulaziz Road, P.O Box 1988, Najran, Kingdom of Saudi Arabia; 8 Department of Civil Engineering, College of Engineering, Northern Border University, 73222 Arar, Saudi Arabia; 9 Department of Mechanical Engineering, College of Engineering, University of Ha’il, 81451 Ha’il City, Saudi Arabia; 10 Laboratoire de Mécanique, Matériaux Et Procédés LR99ES05, Ecole Nationale Supérieure d’Ingénieurs de Tunis, Université de Tunis, 5 Avenue Taha Hussein, 1008 Montfleury, Tunis Tunisia; 11 Department of Mechanical Engineering, Isfahan University of Technology, Isfahan, Iran

Correction to: *Scientifc Reports* 10.1038/s41598-024-81044-3, published online 27 November 2024

The original version of this Article contained errors in the Affiliations.

In the original version of this Article Tao Hai was incorrectly affiliated with ‘State Key Laboratory of Public Big Data, Guizhou University, Guizhou Guiyang 550025, China’ and ‘School of Computer and Information, Qiannan Normal University for Nationalities, Duyun, Guizhou 558000, China’.

The correct affiliations are listed below.

Artificial Intelligence Research Center (AIRC), College of Engineering and Information Technology, Ajman University, P.O. Box: 346, Ajman, United Arab Emirates.

Faculty of Data Science and Information Technology, INTI International University, 71800, Malaysia.

The original Article has been corrected.

